# Impact of *fleQ* Deficiency on Resource Allocation and Heterologous Gene Expression in *Pseudomonas putida* Across Various Growth Media

**DOI:** 10.1111/1751-7915.70054

**Published:** 2024-11-21

**Authors:** Junyoung Kim, Sooyeon Lee, Alexander P. S. Darlington, Juhyun Kim

**Affiliations:** ^1^ School of Life Sciences BK21 FOUR KNU Creative BioResearch Group, Kyungpook National University Daegu Republic of Korea; ^2^ Warwick Integrative Synthetic Biology Centre, School of Engineering University of Warwick Coventry UK

**Keywords:** flagella, growth law, *Pseudomonas putida*, resources

## Abstract

*Pseudomonas putida* is widely used in industrial applications, including the recombinant proteins production, because of its natural advantageous properties. In this study, the gene encoding FleQ, the primary regulator of flagellar synthesis, was deleted to construct a new non‐motile *P. putida* KT2440‐derived strain (*ΔfleQ*). The non‐motile cells showed reduced biofilm formation and enhanced expression of a heterologous gene in nutrient‐rich media compared with the wild‐type (WT) strain, attributed to the reallocation of cellular resources from flagellar synthesis and cellular motility. Additionally, the *ΔfleQ* strain exhibited enhanced tolerance to chloramphenicol, indicating higher ribosome production, confirmed by a higher RNA/protein ratio relative to the WT. While the WT strain showed decreased growth and a three‐fold increase in reporter gene activity in minimal media, the *ΔfleQ* strain maintained consistent reporter gene expression and exhibited a relatively higher growth rate. This suggests that the FleQ is involved in modulating proteome allocation based on nutrient quality. The removal of FleQ allows for more flexible resource allocation, creating a chassis strain with nutrient quality‐independent gene expression capacity, which could be valuable in industrial applications where consistent output is essential.

## Graphical abstract

Deletion of the flagella regulator gene (*fleQ*) results in more consistent heterologous gene expression without significantly affecting bacterial growth, potentially due to better resource allocation. This feature allows the *ΔfleQ* strain to produce target proteins predictably and stably across different culture conditions.

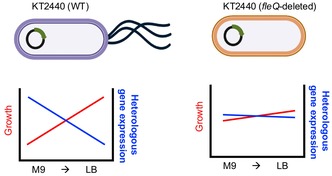

## 1 Introduction

Bacterial gene expression consumes significant cellular resources, which are inherently limited within a cell. Consequently, synthetic circuits designed for heterologous expression often lead to reduced cellular growth (Kotte et al. 2014; Kim et al. 2020, 2021). To cope with these challenges, cells must optimally allocate resources to adapt to diverse stress conditions, including nutrient limitation (Goelzer et al. 2015). For instance, *Escherichia coli* modulate ribosome production in respond to nutrient quality, by enhancing ribosome concentration under nutrient‐rich conditions (Scott et al. 2010; Dai et al. 2016; Serbanescu, Ojkic, and Banerjee 2020). Conversely, in nutrient‐poor media, *E. coli* lowers ribosome levels and re‐allocate resources towards higher production of metabolic enzymes (Harvey 1973; Scott et al. 2014). It has been shown that cellular resources can be reallocated based on the level of nutrient quality, as described by *the growth laws* (Scott et al. 2010; Scott and Hwa 2011). This concept highlights how bacterial proteomes dynamically allocate resources across cellular functions in response to varying growth conditions, optimising growth through adjustments in synthesis of ribosomes and other proteins (Scott et al. 2010, 2014; Scott and Hwa 2011). Leveraging this natural cellular trait, several strategies have been employed to optimise resource allocation to maximise synthetic/heterologous protein expression. One approach to achieve this is genome minimization, where non‐essential genes are removed from the host, facilitating more gene expression machinery, including RNA polymerase and ribosomes, to be allocated to the heterologous genes (Lieder et al. 2015; Calero and Nikel 2019; Lastiri‐Pancardo et al. 2020). Alternatively, reducing resource consumption by recombinant genes ensures more predictable control of gene expression and reduces cellular burden (Martinez‐Garcia, Nikel, Aparicio, et al. 2014; Ceroni et al. 2015, 2018). However, both strategies require knowledge of regulatory networks and essential genes, limiting their application to well‐characterised model organisms (Deng et al. 2011).

To develop bacteria chassis with key industrial applications, inhibiting the expression of host genes that consume substantial cellular resources is an efficient approach. A prime candidate for this is the gene regulating flagellar synthesis. The primary function of cellular flagella is motility, allowing bacteria to swim towards favourable environments and evade harmful conditions (Merino, Shaw, and Tomas 2006). It has secondary roles in biofilm formation, adherence and pathogenesis (Duan et al. 2013; Haiko and Westerlund‐Wikstrom 2013). Recent findings indicate that the number of flagella per cell remains constant regardless of nutrient conditions (Honda et al. 2022). However, this energy‐consuming motility apparatus (Berry and Armitage 1999; Martinez‐Garcia, Nikel, Chavarria, et al. 2014) is unnecessary in the nutrient‐rich conditions of a biomanufacturing fermentation process. Thus, inactivating flagellar synthesis could increase heterologous gene expression capacity, by conserving cellular resources (Lieder et al. 2015; Volke and Nikel 2018). The gene *fleQ* is the primarily regulator of flagellar synthesis (Kieboom et al. 2001; Capdevila et al. 2004; Redondo‐Nieto et al. 2008; Bush and Dixon 2012; Jimenez‐Fernandez et al. 2016). Recently several research groups have used adaptive laboratory evolution of *P. putida* to enhance biomanufacturing performance and have identified emergent mutations in the *fleQ* gene of strains with enhanced metabolic activities (Mueller et al. 2022), coumaric acid tolerance (Mohamed et al. 2020) and biomass yield (Al‐Tameemi and Rodriguez‐Verdugo 2024).

Structurally, FleQ is a member of the AAA + ATPase family of proteins and features a C‐terminal helix‐turn‐helix DNA binding domain (Bush and Dixon 2012; Molina‐Henares et al. 2017). It also has a domain characteristic of proteins that interact with the sigma factor σ^54^ (Bush and Dixon 2012; Molina‐Henares et al. 2017) and homologous genes are found in diverse bacteria (Spohn and Scarlato 1999; Kim and McCarter 2000; Homma et al. 2022; Schwan et al. 2022). These structural features enable FleQ to interact with c‐di‐GMP and bind to the promoters of flagella and exopolysaccharide synthesis genes in *P. putida* (Molina‐Henares et al. 2017). In this study, a derivative strain of *P. putida* KT2440 was generated by deleting the *fleQ* gene, which regulates genes associated with flagellar formation, motility, adhesion, and exopolysaccharide production (Blanco‐Romero et al. 2018; Leal‐Morales et al. 2022). The *ΔfleQ* strain exhibited higher reporter gene expression than the wild‐type (WT) when grown in rich medium. In contrast, the WT strain exhibited a less vigorous growth in comparison to the *ΔfleQ* derivative, accompanied by enhanced heterologous protein synthesis, suggesting that *P. putida* KT2440 adheres to the previously described growth laws (Klumpp, Zhang, and Hwa 2009; Scott et al. 2014; Maitra and Dill 2015). Interestingly, the *ΔfleQ* strain sustained higher maximum growth than the WT, even in minimal media. Additionally, the gene expression capacity of the *ΔfleQ* strain remained constant across different nutrient conditions, implying an alternative adaptive strategy compared to the WT. These findings suggest that deleting *fleQ* could serve as a simple strategy for developing new microbial chassis using organisms of key industrial interest. This approach could lead to more predictable and stable protein production, simplifying process control and improving efficiency in biotechnological applications, particularly in large‐scale processes where consistent output is crucial.

## 2 Results and Discussion

### 2.1 Deletion of *fleQ* Results in Loss of Motility and Reduced Biofilm Formation

**FIGURE 1:**
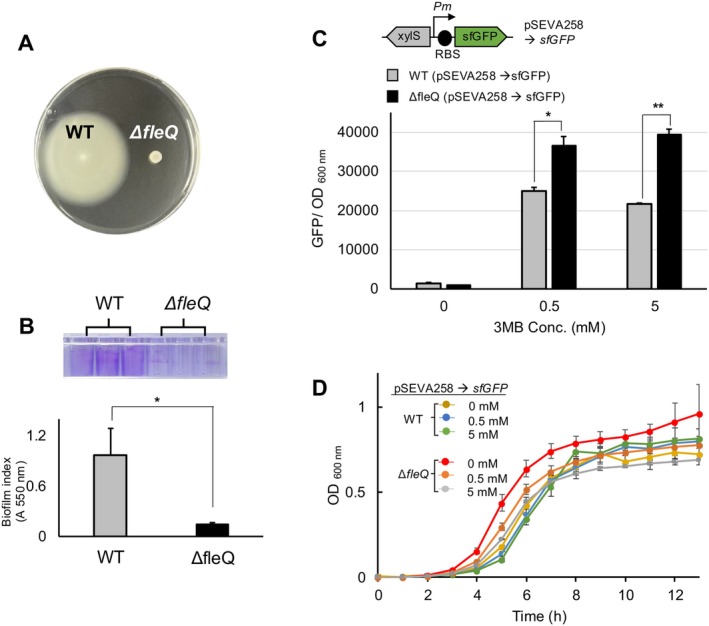
Comparative analysis of *P. putida* KT2440 WT and *ΔfleQ* strains based on motility, gene expression capacity, and growth characteristics. (A) To assess bacterial swimming, a single colony grown in LB was resuspended in 5 μL of the same culture, and the sample was spotted onto 0.3% (w/v) LB agar plates and incubated overnight at 30°C. (B) Biofilm formation was quantified with crystal‐violet (CV) solution as described in Section 4. The staining result is shown in the upper panel. The biofilm index is represented as the ratio of CV staining (*A*_550 nm_) to planktonic cell density (CV/ OD_600 nm_). Student's *t*‐test analysis revealed significant differences in biofilm formation between the two strains (*p ≤ 0.05). (C) Strains carrying the pSEVA258‐SfGFP plasmid were used to express the reporter protein, with 0.5 or 5 mM of 3‐methyl benzoate (3MBz) added to the media. The strains were incubated for 13 h at 30°C in a microplate reader. The expression levels of the fluorescent reporter were normalised to cell density and the maximum intensities of sfGFP at the 13‐h time point. *p, **p ≤ 0.005 (Student's *t*‐test). (D) The growth profiles of the strain are shown. Error bars represent means ± SD (*N* = 3).

Both the production and utilisation of flagella, which consists of 59 proteins, are energetically costly for *P. putida* (Berry and Armitage 1999; Colin et al. 2021; Leal‐Morales et al. 2022). Engineering non‐motile cells can conserve cellular resources and allocate them to genes, making such cells ideal for use as microbial cell factories. More than 50 genes have been reported to be involved in the synthesis of flagella with four levels of cascade regulation in *P. putida* (Jimenez‐Fernandez et al. 2016). The product of *fleQ* acts as a transcriptional regulator controlling the expression of these genes (Arora et al. 1997; Blanco‐Romero et al. 2018). In the present study, a *fleQ* deleted strain was generated by deleting the gene encoding this master regulator using a seamless allelic replacement method in the *P. putida* KT2440 strain (Martinez‐Garcia and de Lorenzo 2011). The resulting *ΔfleQ* strain did not exhibit swimming motility on soft agar plates (Figure 1A). Additionally, it showed reduced biofilm formation compared to the WT, as determined using a crystal‐violet (CV) assay (O'Toole 2011), which showed an approximately seven‐fold higher CV staining value in the WT relative to the mutant (Figure 1B). This reduction in biofilm formation is due to the inactivation of *fleQ*, which decreases the activation of genes associated with biofilm formation, such as *lapA* and *bcs*, which encode crucial components of the biofilm matrix (Martinez‐Gil, Ramos‐Gonzalez, and Espinosa‐Urgel 2014; Xiao et al. 2016). Given the consistent phenotype of the non‐flagellated cells (Martinez‐Garcia, Nikel, Chavarria, et al. 2014; Blanco‐Romero et al. 2018), our observations indicate that the *ΔfleQ* strain does not express genes associated with flagella synthesis due to the loss of the master regulator. We then used this strain to investigate how the loss of motility affects the gene expression capacity.

### 2.2 The *ΔfleQ* Strain Exhibits Enhanced Heterologous Genes Expression Under the Nutrient‐Rich Condition

A reporter plasmid carrying the superfolder green fluorescent protein (*sfGFP*) gene (Pédelacq et al. 2006) inducible by 3‐methylbenzoate (3MBz) (Martinez‐Garcia et al. 2015; Gawin, Valla, and Brautaset 2017; Volke et al. 2020) was constructed. The resulting plasmid, pSEVA258‐sfGFP, was introduced into both the WT and *ΔfleQ* strains (Figure 1C). The reporter cells were incubated in a 24‐well microplate containing culture media at 30°C, and the fluorescent activity was monitored every hour for 13 h using a microplate reader. The peak fluorescence signal at the 13‐h time point was used to assess the gene expression capacity of the cells. When the reporter activity was measured in cells grown in Luria–Bertani (LB) medium with 0.5 mM of 3MBz, enhanced sfGFP signals appeared in both strains (Figure 1C). However, increasing the inducer concentration to 5 mM of 3MBz did not noticeably affect the maximum level of the reporter expression (Figure 1C). The *ΔfleQ* strain exhibited a 1.5‐fold higher accumulation of sfGFP compared to the WT when cultured with 0.5 mM of 3MBz (Figure 1C). Importantly, the growth of both strains was comparable under these conditions (Figure 1D), indicating that the higher GFP activity in the mutant was not due to larger population size. This observation led us to hypothesise that the *ΔfleQ* strain carried more available cellular resources, such as ATP or ribosomes, potentially contributing to enhanced protein synthesis capacity. To confirm this, we quantified the intracellular ATP levels in cells that do not carry the reporter, grown in either LB or M9 minimal media with a single carbon source, such as succinate or glucose, during mid‐exponential phase. This was done using an ATP bioluminescence kit and a microplate reader (see Section 4). However, the results of the ATP assay indicated no significant difference in ATP levels between the WT and *ΔfleQ* strains (Figure S1).

**FIGURE 2:**
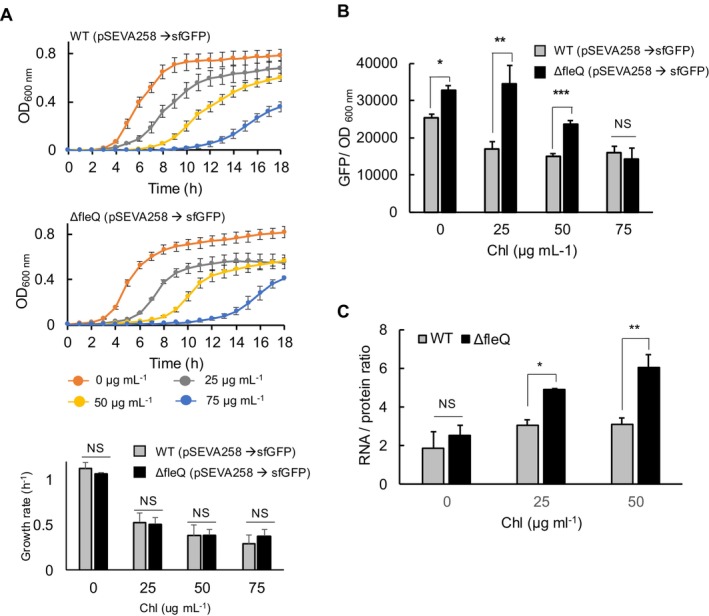
Impact of partial translation inhibition on growth and reporter activity. (A) Overnight cultures of both the WT and *ΔfleQ* strain carrying the reporter plasmid were diluted 1:500 into LB medium within wells of a 24‐well microplate. The media were supplemented with 0.5 mM of 3MBz and varying concentrations of chloramphenicol (Chl; 0, 25, 50 and 75 μg mL^−1^). Subsequently, cells were incubated at 30°C for 18 h in the microplate reader. Growth rates were determined by taking natural logarithms of OD_600nm_ and fitting a straight line to the linear portion of the growth profile. Cellular growth gradually decreased with increasing Chl concentration. (B) Fluorescence activities at the 18‐h time point for each experimental condition were recorded. Error bars represent means ± SD (*N* = 3). *p, **p, ***p ≤ 0.05 (Student's *t*‐test). (C) The RNA/ protein ratio was calculated to determine ribosome abundance in the two strains cultivated in LB supplemented with Chl. Error bars represent means ± SD (*N* = 3). *p, **p ≤ 0.05 assessed using Student's *t*‐test.

These results suggested that the increased availability of cellular resources, such as ribosomes, could contribute to enhanced reporter activity. To explore this further, we partially inhibited translation using chloramphenicol (Chl); (Bulkley et al. 2010; Volkov, Seefeldt, and Johansson 2019). Note that *P. putida* strains exhibit a higher natural resistance to Chl compared to other bacteria, resulting in sub‐lethal concentrations that are higher than what might be expected (Fernandez et al. 2012). When the reporter cells were cultured in LB medium supplemented with both the inducer (3MBz; 0.5 mM) and Chl (25, 50 and 75 μg mL^−1^), a gradual inhibition of growth was observed in both WT and *ΔfleQ* strains (Figure 2A). As the Chl concentration increased, growth inhibition became more pronounced, leading to a decrease in growth rate (Figure 2A). Interestingly, the *ΔfleQ* strain showed similar levels of fluorescence signals even after treatment with 25 μg mL^−1^ Chl, whereas the WT strain exhibited lower expression levels of the reporter (Figure 2B). Moreover, at 50 μg mL^−1^ Chl, the *ΔfleQ* strains accumulated a higher amount of the fluorescent protein compared to the WT (Figure 2B). Additionally, the *ΔfleQ* strain demonstrated increased resistant to Chl (Figure S2), although there was no difference in GFP expression activity between the two strains at the highest Chl concentration tested (Figure 2B). To confirm that the increased tolerance in the *ΔfleQ* strain is attributed to higher ribosome abundance, we measured the RNA‐to‐protein (R/P) ratio. This method allows quantification of ribosome levels, as the protein content remains relatively constant in growing cells, while the number of ribosomes correlates with the amount of rRNA, the predominant component of total RNA (Neidhardt and Magasanik 1960; Ecker and Schaechter 1963; Bremer and Dennis 2008; Scott et al. 2010; Matamouros et al. 2023). The ratio reflects how the cell allocates its resources between building the machinery needed for ribosomes and the synthesis of other proteins necessary for various cellular functions; high growth rates require a substantial investment in ribosome production, leading to a higher R/P ratio (Scott et al. 2010). To measure the R/P ratio, cells in the exponential growth phase in LB supplemented with Chl were prepared, and both total RNA and proteins were isolated. Analysis of the R/P ratio revealed a higher ribosome content in the *ΔfleQ* strain compared to the WT under Chl treatment although we were unable to harvest samples at the 75 μg mL^−1^ Chl condition due to low cellular fitness (Figure 2C). These findings suggest that the *ΔfleQ* strain exhibits reduced sensitivity to translational inhibition due to its increased capacity to produce ribosomes. It is also worth to note that we observed reduced fluorescent signals at 50 μg mL^−1^ Chl (Figure 2B). However, the higher R/P ratio was observed under the same condition in the *ΔfleQ* strain (Figure 2C). This result indicates that higher ribosome content does not always mean increased heterologous gene expression. Instead, the reduced heterologous gene expression is a consequence of the altered proteome partitioning within the strain, where most ribosomes are being allocated to the ribosome‐associated fraction. According to bacterial growth laws, this shift in proteome allocation reflects an adaptation to prioritise essential cellular functions over heterologous protein synthesis, resulting in lower expression levels under the tested conditions (Scott et al. 2010; Scott and Hwa 2011). In contrast, in the absence of Chl, there was no significant difference in the R/P ratio between the two strains (Figure 2C), even though the *ΔfleQ* strain exhibited higher GFP accumulation (Figure 2B). This suggests that the *ΔfleQ* strain adopts a different resource allocation strategy compared with the WT, despite having similar ribosome levels. Therefore, disabling flagellar synthesis reallocated resources in a way that enabled the *ΔfleQ* strain to boost GFP expression and enhance resistance to translation inhibition.

Since the increased gene expression capacity observed in the *ΔfleQ* strain was achieved at the expense of flagellar synthesis under the nutrient‐rich condition, we also investigated whether this cost‐saving effect persisted in minimal media.

### 2.3 Growth Profile and Gene Expression Capacity of the *ΔfleQ* Strain in Defined Media

Under nutrient‐limited conditions, cells, such as those of *E. coli* , primarily adopt a strategy of downregulating ribosome synthesis (Scott et al. 2014; Kafri et al. 2016). This promotes the reallocation of cellular resources towards essential metabolic processes, particularly those involved in amino acid production (Scott and Hwa 2011). While this adaptation allows cells to maintain vital functions, it results in reduced cellular growth (Scott et al. 2014; Kafri et al. 2016). Given that the *ΔfleQ* strain exhibited higher GFP expression activities in a nutrient‐rich medium (LB) compared to the WT, we sought to understand how the mutant strain balances cellular growth and gene expression under nutrient‐poor conditions, which generally decrease cellular growth as described by the growth law. To explore this, reporter strains were cultured in M9 minimal medium supplemented with either succinate (0.2%) or glucose (0.2%) as a single carbon source, and their fluorescent signals were measured.

**FIGURE 3:**
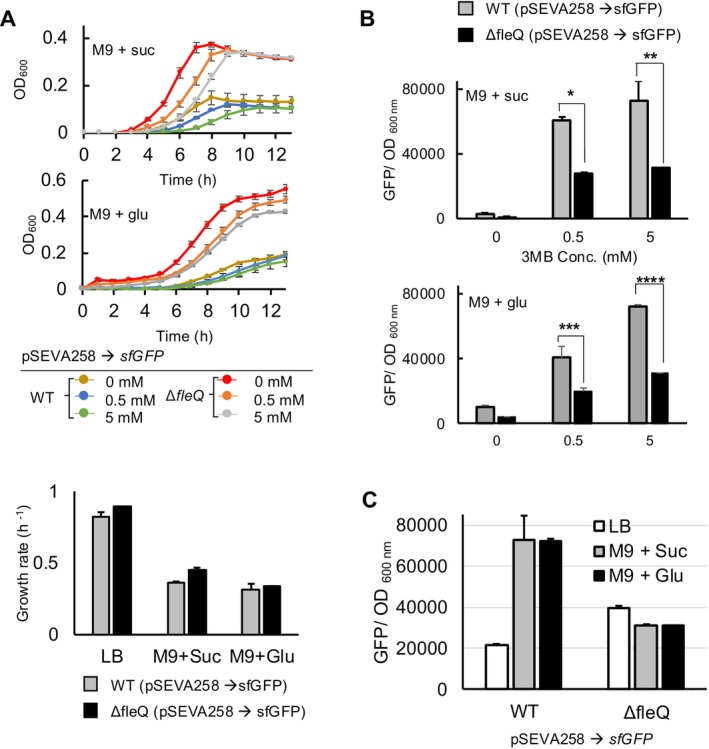
Low nutrient quality does not influence growth‐dependent resource allocation in the *ΔfleQ* strain. (A) The reporter strains were cultured in M9 minimal media containing either succinate (0.2%) or glucose (0.2%) and 0, 0.5, or 5 mM of 3MBz, and the growth of both strains was monitored for 13 h. The growth rates of each strain were determined from the growth profiles shown in Figure 1D (for LB medium) and Figure 3A (for minimal media) under conditions of treatment with 5 mM of 3MBz. (B) The graph shows normalised reporter intensities at the 13‐h time point, measured under culture conditions identical to those described in panel (A). *p, **p, ***p, ****p ≤ 0.05 (Student's *t*‐test). (C) Comparison of the fluorescence activities across different growth conditions. sfGFP signals were acquired from cell cultures treated with 5 mM of 3MBz. Contextual reference data from both LB and M9 + succinate/ glucose conditions were integrated from Figures 1C and 3B respectively. Error bars represent means ± SD (*N* = 3).

The cell growth rate was decreased in both strains when cultured in minimal media supplemented with either succinate (0.2%) or glucose (0.2%; Figure 3A) compared to that in LB (Figure 1D). Although the lag time for both strains increased with the addition of 3MBz at all concentrations tested, this did not affect the maximum growth level (Figure 3A). The WT strain exhibited a more pronounced reduction in cellular growth, measured based on maximum optical density at 600 nm (OD_600 nm_) at 13 h, and a greater increase in GFP expression level compared to the *ΔfleQ* strain when grown in minimal media (Figure 3A,B). The fluorescent signals obtained from the culture with 5 mM of 3MBz increased three‐fold when the WT was grown in minimal media compared to when it was cultured in LB (Figure 3C). The negative correlation between maximal cell proliferation and gene expression capacity can be explained by nutrient‐dependent proteome allocation (Serbanescu, Ojkic, and Banerjee 2020). Bacteria allocate limited resources between growth and protein synthesis. In nutrient‐rich conditions, more resources go towards growth, reducing gene expression. In nutrient‐limited conditions, bacteria shift resources to protein synthesis, enhancing gene expression but slowing growth. This trade‐off allows bacteria to adapt their proteome to varying environmental conditions. However, the *ΔfleQ* strain does not exhibit the same nutrient‐dependent effects. Similar fluorescent signals were detected in the *ΔfleQ* strain across all examined media, indicating consistent proteome allocation regardless of growth conditions (Figure 3C). This consistency explains why the *ΔfleQ* strain showed a higher maximum OD_600 nm_ in minimal media compared to the WT (Figure 3A); the *ΔfleQ* strain allocates more resources to the ribosome‐associated fraction, resulting in less reporter protein accumulation due to the finite resource pool in the cell (Figure 3C). Given that the reporter system is driven by the XylS‐Pm expression module, the increased expression observed in minimal media could be due to the release of repression by host factors, particularly the Crc (Catabolite Repression Control) global regulator (Moreno, Fonseca, and Rojo 2010). In order to test if catabolic repression released would be involved in higher levels of GFP expression observed in defined media, we employed an orthogonal isopropyl β‐D‐1‐thiogalactopyranoside (IPTG) expression system based on LacIq‐Ptrc promoter, to induce the reporter gene using the pSEVA234 plasmid. The same expression pattern was observed: fluorescent signals increased in minimal media supplemented with a sole carbon source (Figure S3), suggesting that catabolic repression triggered by rich medium was not the cause. These findings indicate that neither the cell's metabolic status (carbon source) nor the host factor is associated with determining the gene expression capacity of the *ΔfleQ* strain. Instead, the growth rate inversely influences the production of the reporter, but this effect is only evident in the WT strain (Figure 3). We also modified the reporter plasmid to carry the RK2 origin of replication (Doran, Konieczny, and Helinski 1998), enabling the cell to maintain the plasmid at a low copy number. This reduction in gene dosage had no impact on the resource allocation strategy as the *ΔfleQ* strain consistently displayed the same level of sfGFP regardless of culture conditions. In contrast, the WT cells exhibited increased fluorescent signals with a dramatic reduction in the maximum OD_600 nm_ in response to changes in nutrient quality (Figure S4). Therefore, we suggested that *ΔfleQ* strain allocates a higher proportion of their cellular resources to ribosomal‐associated fractions compared to the WT when the nutrients are scarce. This strategy enables the strain to maintain its growth rate but it comes at the cost of a consistent gene expression capacity that remains unaffected by varying culture conditions.

Thus, by conserving cellular resources instead of investing in flagellar synthesis, the cell can allocate its proteomes across various cellular components regardless of nutrient conditions. This enables the *fleQ*‐deleted derivative to serve as a synthetic biology chassis for cost‐effective, large‐scale production of heterologous protein expression in industrial settings, where consistent and reliable output is essential.

### 2.4 Overexpression of FleQ Leads to Decreased Cellular Fitness

**FIGURE 4:**
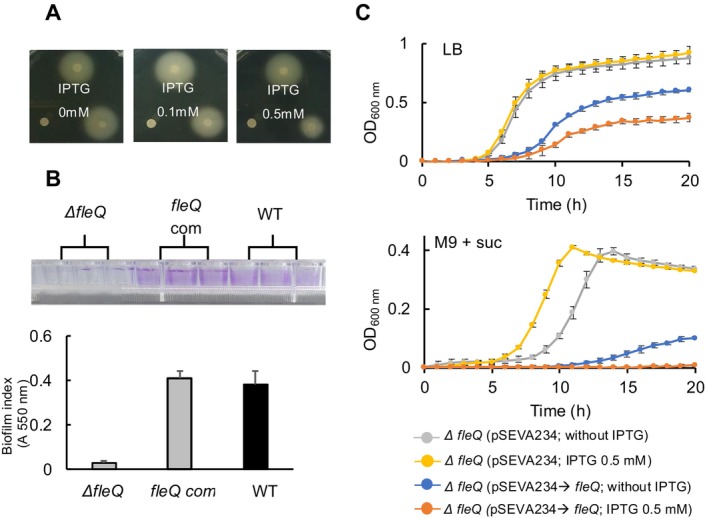
Influence of FleQ overexpression on maximum growth and growth rate. The *fleQ* gene was cloned into pSEVA234, which features an IPTG‐dependent inducible system. Swimming assays (A) and biofilm formation analyses (B) were performed to assess the complementation of FleQ. (C) Cells containing the FleQ‐expression plasmid were cultured in LB or succinate‐amended minimal media with 0.5 mM IPTG added to induce the regulator gene. Additionally, the same experiments were conducted with strains harbouring the empty vector. Error bars represent means ± SD (*N* = 3). ‘*fleQ* com’ refers to fleQ complementation.

To confirm our findings and further characterise the effect of deleting the *fleQ* gene on resource allocation, we overexpressed FleQ in the *ΔfleQ* strain. To achieve this, *fleQ* with a strong ribosome‐binding site was cloned into pSEVA234, a plasmid carrying the IPTG inducible *LacI* ^*q*^ *‐Ptrc* expression system (Martinez‐Garcia et al. 2023). Introduction of the resulting plasmid, pSEVA234‐*fleQ*, into the *ΔfleQ* strain, restored the strain's cellular motility on soft agar (Figure 4A), even in the absence of the inducer due to the leaky expression of the inducible system. Consistent with the restored phenotype, the recombinant plasmid facilitated biofilm formation in the *ΔfleQ* strain when cultured in LB with IPTG (0.5 mM). Furthermore, the CV analysis demonstrated comparable biofilm formation value between the WT and the *ΔfleQ* strains harbouring the *fleQ*‐expressing plasmid, while the *ΔfleQ* strain without the complementary plasmid exhibited only basal level of biofilm formation (Figure 4B). Examining the growth dynamics of the complemented strain in LB revealed that cellular growth was influenced by the level of FleQ expression, since the pSEVA234 backbone‐containing cells did not show reduced growth (Figure 4C). Retarded cellular growth was observed in the presence of IPTG, although the inducer did not affect the growth of the *ΔfleQ* strain (Figure 4C). This suggests that high levels of accumulated FleQ may lead to lower allocation of cellular resources to the ribosome‐associated fraction, which is crucial for supporting cellular growth (Scott et al. 2010). In accordance with this scenario, overexpression of FleQ led to a decrease in both growth rate and the maximum growth achieved OD in succinate supplemented minimal medium (Figure 4C). Interestingly, IPTG treatment resulted in a longer lag phase in the *ΔfleQ* strain, although neither the growth rate nor maximum growth were significantly affected (Figure 4C).

Previous studies have reported that the FleQ overexpression can lead to excessive flagellar synthesis, potentially resulting in an increased number of flagella per cell (Kutsukake and Iino 1994; Kearns and Losick 2005; Aldridge et al. 2010; Erhardt and Hughes 2010). Such non‐optimal flagella numbers could cause the cell to expend more energy and allocate cellular resources inefficiently (Fung and Berg 1995; Gabel and Berg 2003; Minamino et al. 2014). Moreover, the role of FleQ extends beyond the regulation of the motility machinery; as a master regulator in *Pseudomonas* species, it also governs genes involved in diverse cellular processes, including iron homeostasis, cell wall formation, c‐di‐GMP metabolism, transport, and virulence in *Pseudomonas* species (Blanco‐Romero et al. 2018). Chromatin immunoprecipitation sequencing (ChIP‐seq) analysis has identified 103 putative FleQ binding sites within the *P. putida* genome (Blanco‐Romero et al. 2018), highlighting its broad regulatory influence. This suggests that the reallocation of cellular resources in the *ΔfleQ* strain might be influenced by factors beyond flagellar synthesis. To explore whether cellular resource allocation is solely influenced by flagellar synthesis, rather than by other factors regulated by FleQ, we employed the KT2440 Δflagella strain, which lacks the *fleQ* gene along with key components essential for flagellar export, assembly, and chemotaxis (Martinez‐Garcia, Nikel, Chavarria, et al. 2014). Introducing the pSEVA234‐fleQ plasmid into both the Δflagella and WT strains alongside a reporter plasmid allowed us to assess gene expression capacity. The pSEVA658‐sfGFP plasmid carrying gentamycin resistance was employed to maintain both plasmids in the cells during analysis. In LB supplemented with inducers (IPTG, 0.1 mM; 3MBz, 5 mM), both the growth profiles and reporter activities closely mirrored those observed in the *ΔfleQ* strain (Figure S5A). While there were minor differences in growth profiles, the non‐flagellated strain (Δflagella) exhibited a 1.5‐fold higher GFP expression level compared to the WT. Similarly, in the M9 minimal medium supplemented with succinate and the same inducers, the Δflagella strain displayed superior cellular fitness, while the WT strain showed 3‐fold higher fluorescent signals compared to the Δflagella strain (Figure S5B). These findings align with our observations in the *ΔfleQ* strain, indicating that while FleQ plays a pivotal regulatory role, the absence of flagellar synthesis remains crucial in reallocating cellular resources depending on culture conditions.

Several *Pseudomonas* species have already been engineered to enhance gene expression and biomass yield by removing non‐essential genes including flagellar synthesis genes (Martinez‐Garcia, Nikel, Aparicio, et al. 2014; Wynands et al. 2019). While these strains are available as chassis strains, further engineering is required for other species with potential for specific applications. In this study, we demonstrated that removing a single global regulator that activated unneeded proteins in *P. putida* , can consistently allocate resources to heterologous genes, maintaining a similar level of distribution regardless of culture conditions. This approach offers a promising strategy for rapidly engineering diverse strains with significant biotechnological applications.

## 3 Conclusion

Herein, we demonstrated that deleting *fleQ* in *P. putida* reduces the energy‐intensive production of flagellar proteins and cellular motility, thereby increasing the capacity for heterologous gene expression. This effect is particularly pronounced in nutrient‐rich conditions, as the cells allocate more cellular resources towards non‐ribosomal genes. The *ΔfleQ* strain used in this study distributed cellular proteome independently of growth rate, maintaining heterologous expression across diverse media. This unique characteristic is attributed to the moderate modulation of gene expression capacity, which maximises cellular growth even in a defined medium. In contrast, the WT strain displayed a three‐fold increase in reporter activity when transitioning to lower‐quality culture conditions, albeit with a concomitant reduction in growth rate. Notably, the global regulator, FleQ, not only governs flagellar synthesis but also influences a multitude of other genes, including those involved in iron metabolism and signal transduction pathways. Nevertheless, these additional functions are not vital for basic cellular growth and gene expression. The removal of this regulator, leading to the absence of flagella, allows the cell to adopt a distinct resource allocation strategy compared with the WT. This valuable trait positions this strain as a promising chassis in synthetic biology applications, particularly within the context of *Pseudomonas* species. The methodology and insights derived from this study demonstrate that targeted manipulation of cellular resources through the deletion of key regulators is an essential approach for achieving stable resource allocation to heterologous genes, ensuring consistent expression across diverse culture conditions. This strategy offers a rapid and promising pathway for engineering bacterial strains with significant biotechnological potential.

## 4 Materials and Methods

### 4.1 Strains, Plasmids and Growth Conditions

Bacterial strains and plasmids used in this study are listed in Table S1. *E. coli* and *P. putida* were routinely grown at 37°C and 30°C, respectively, in LB (BD, NJ, USA) or M9 minimal medium (Sigma‐Aldrich, St. Louis, MO, USA) supplemented with either succinate (0.2%; w/v) or glucose (0.2%; w/v) as the sole carbon source. When required, antibiotics were added to the culture media to maintain the plasmids used in this study. The antibiotics and their concentrations were as follows: kanamycin (Km, 50 μg mL^−1^), ampicillin (Ap, 150 μg mL^−1^) or gentamicin (Gm, 20 μg mL^−1^). To assess ribosome abundance in response to antibiotic stress, cells were exposed to varying concentrations of chloramphenicol (Chl; 0, 25, 75 and 175 μg mL^−1^). To monitor growth and gene expression profiles, a single colony from the strain of interest was taken from a fresh plate and cultured overnight in 1 mL of liquid medium. The overnight cultures were then diluted 100‐fold in 1 mL of the same medium in 24‐well plates, and growth and fluorescence were monitored using a microplate reader (Synergy H1, BioteK, CA, USA). To induce sfGFP reporter protein expression, 3MBz (Sigma‐Aldrich, St. Louis, MO, USA) was added to cultures at final concentrations of 0.5 and 5 mM. Optical density at 600 nm and green fluorescent signals (excitation: 485 nm, emission: 525 nm) were recorded at 60‐min intervals.

### 4.2 Cloning Procedures and Construction of Reporter Strains

The bacterial strains, plasmids and primers used for constructing the reporter strains are described in Tables S1 and S2. Standard protocols for DNA handling followed (Chong 2001). Plasmid DNA was isolated from bacterial cells using a plasmid DNA purification kit (Exprep Plasmid SV, Geneall, Seoul, Korea). The seamless allelic replacement method described by Martinez‐Garcia and de Lorenzo (2011) was used to construct non‐flagella‐producing cells from *P. putida* KT2440. The delivery plasmid carrying the flanking regions of the *fleQ* gene was constructed as follows. First, the upstream (*TS1‐fleQ*, ~0.5 kb) and downstream (*TS2‐fleQ*, ~0.5 kb) regions of *P. putida* around *fleQ* were amplified using the primer pairs fleQ‐TS1F/R and fleQ‐TS2F/R respectively (Table S2). Subsequently the PCR fragments were linearly assembled using overlap extension PCR, and cloned into the pEMG vector (Table S1), which was digested with BamHI and HindII yielding pEMG‐fleQTS1‐TS2. This plasmid was maintained in *E. coli* SY327 λpir and subsequently transferred to *P. putida* KT2440 via electroporation. The cointegration was confirmed using colony PCR. Next, the pSW plasmid harbouring the *I‐SceI* endonuclease (Wong and Mekalanos 2000; Martinez‐Garcia and de Lorenzo 2011) and the Pm promoter was introduced via electroporation into the cointegrated strain, which subsequently had both Km and Ap resistance. Clones were grown in LB medium with Ap (500 μg mL^−1^) and 3MBz (5 mM) to activate the Pm promoter, allowing *I‐SceI* expression. Cells were plated on LB agar and screened for the deletion of *fleQ* by selecting Km‐sensitive clones. Once the deletion was confirmed, pSW was finally cured via serial dilutions in the absence of antibiotics, yielding strain *P. putida* *Δ* *fleQ* strain.

To generate a reporter plasmid carrying the sfGFP gene, the gene was amplified from pET28b‐sfGFP using the primer pairs sfgfp‐F/−R (Table S2). The resulting amplicon was cloned into the HindIII and BamHI sites of pSEVA228, pSEVA258 and pSEVA234, resulting in the constructs pSEVA228‐sfGFP, pSEVA258‐sfGFP and pSEVA234‐sfGFP respectively. To activate the reporter gene, either 3MBz (0.5 or 5 mM) or isopropyl β‐D‐1‐thiogalactopyranoside (IPTG; 1 mM) was added into culture media. Using the same approach, pSEVA234‐fleQ was constructed using primer pairs fleQ‐comF/−R and introduced into the *ΔfleQ* strain. To express *fleQ*, the cells were cultured with IPTG (Sigma‐Aldrich; 0.1 or 0.5 mM).

### 4.3 Swimming and Biofilm Assay

To assess motility, both the WT and *ΔfleQ* strain were cultured overnight in LB. Each culture was then diluted 100‐fold in LB, and 5 μL of each sample was spotted onto LB agar plates containing 0.3% (w/v) agar. The plates were incubated overnight at 30°C, and the diameter of the swimming halo was measured. Biofilm formation was quantified using the CV microtiter dish assay, as described previously (O'Toole 2011). The WT and *ΔfleQ* strains were incubated overnight at 30°C with continuous shaking in a 96‐well microplate. Upon reaching maximum OD 600_nm_, the samples were discarded, and the plate was washed twice with distilled water, followed by the addition of 125 μL of Crystal Violet (CV) solution (0.1% CV (*v/v*) in distilled water), Sigma‐Aldrich. After a 15‐min incubation, the plate was washed three times with distilled water and allowed to dry overnight. To solubilise the CV, a solution of 30% acetic acid (*v/v*) in distilled water was added, followed by another 15‐min incubation. The contents of each well were then transferred to a new 96‐well microplate and the optical density was measured at 550 nm using a Synergy H1 microplate reader.

### 4.4 ATP Bioluminescence Assay

To assess ATP levels in both the WT and *ΔfleQ* strains, we cultured cells in three different media: LB, M9 minimal medium with succinate (0.2%), and M9 minimal medium with glucose (0.2%). The cells were harvested during the mid‐exponential phase, which corresponded to an OD_600nm_ of approximately 0.8 for LB and 0.4 for the minimal media. These samples were then prepared for ATP quantification. The intracellular ATP concentration was measured using an ATP bioluminescence assay kit (ENLITEN ATP Assay System, Promega, WI, USA) by following the manufacturer's protocol. Luminescence signals were recorded using a Synergy H1 microplate reader (Optics type: Luminescence fibre, Gain: 100). The values were converted into nM units using a standard curve and normalised to the OD value.

### 4.5 Measurement of RNA/Protein Ratio

Overnight cultures of *P. putida* KT2440 WT and *ΔfleQ* strains were 100‐fold diluted into 10 mL of LB media. Chl was added to each culture at final concentrations of 0, 25 and 50 μg mL^−1^. The cultures were then incubated with shaking at 30°C until they reached exponential phase (OD_600nm_ ~ 0.8). Subsequently, 1 mL of each culture was prepared for RNA extraction, and another 1 mL was prepared for protein extraction. For RNA protection, the prepped cells were resuspended in an ice‐cold phenol/ethanol solution (5% water‐saturated phenol in ethanol). Cell pellets were then resuspended in a lysis solution (2 mg mL^−1^ lysozyme in Tris‐Cl, pH 7.5) and incubated at 37°C for 10 min to lyse the cells. The resulting solutions (100 μL) were processed using the RNeasy Mini Kit (Qiagen) following the manufacturer's protocols. The RNA concentrations were measured using a Take3 microplate reader (Biotek, CA, USA). For protein extraction, the samples were homogenised using a VCX‐130 PB ultrasonic processor (Sonics & Materials, USA) for a total of 5 min, employing 30‐s pulses on and off at 40% amplitude while kept on ice. After homogenization, the samples were centrifuged to pellet the cell debris. Supernatants (10 μL) were mixed with 300 μL of Coomassie PlusTM Protein Assay Reagent (Thermo Fisher Scientific, MA, USA) and incubated in a 96‐well microplate at room temperature for 10 min. Distilled water served as a blank. To quantify total proteins, a standard curve was prepared using albumin standards (Thermo Fisher Scientific, MA, USA) at final concentrations of 0, 200, 400, 600, 800 and 1500 μg/mL with the same Coomassie reagent. Absorbance was measured at 595 nm using a Synergy H1 microplate reader. All samples were analysed in three biological replicates. The RNA concentration (ng/μL) was divided by protein mass (μg/mL) to calculate the RNA/ protein ratio.

## Author Contributions

**Junyoung Kim:** conceptualization, data curation, formal analysis, investigation, methodology, validation, writing – original draft. **Sooyeon Lee:** data curation, formal analysis, investigation, methodology. **Alexander P. S. Darlington:** conceptualization, validation, writing – original draft, writing – review and editing. **Juhyun Kim:** conceptualization, data curation, formal analysis, funding acquisition, supervision, validation, writing – original draft, writing – review and editing.

## Conflicts of Interest

The authors declare no conflicts of interest.

## Supporting information


Supporting Information S1.

Supporting Information S2.

## Data Availability

The data is available in the manuscript.
