# Supplementary material for: Impact of fleQ Deficiency on Resource Allocation and Heterologous Gene Expression in Pseudomonas putida Across Various Growth Media
Source: Microb Biotechnol. 2024 Nov 21;17(11):e70054. doi: 10.1111/1751-7915.70054 (PMC11580810; doi:10.1111/1751-7915.70054)
Supplement: Supplementary file 1 — Supporting Information S1. [file MBT2-17-e70054-s002.docx]

**Supplementary Table S1. Bacterial strains, plasmids used for this study**

| Strains | Characteristics | Sources |
| --- | --- | --- |
| *Pseudomonas putida* | | |
| KT2440 | Wild type cured of pWW0 | (Nelson et al., 2002) |
| KT2440 Δ*fleQ* | KT2440 with deletion of *fleQ* | This work |
| KT2440 Δ*flagella* | KT2440 derivative, non-flagellated | (Martinez-Garcia et al., 2014) |
| *Escherichia coli* | | |
| DH5α | Strain for cloning; Δ(*lac*ZYA-*arg*F) endA1 gyrA96 hsdR17(rK–, mK+) phoA recA1 relA1 supE44 λ–thi-1 F– φ80lacZΔM15 | (Grant et al., 1990) |
| SY327 λpir | λpir argE(Am) nalA rif recA56 | (Miller and Mekalanos, 1988) |
| Plasmids | | |
| pEMG | Suicide plasmids for deletion of genes, Km^R^, R6K origin, oriT, lacZα with two flanking I-sceI regions | (Martinez-Garcia and de Lorenzo, 2011) |
| pEMG-fleQTS1-TS2 | pEMG plasmid containing flanking regions TS1, TS2 of *fleQ* for deletion of the gene | This work |
| pSW | Plasmid for expression of I-SceI endonuclease, Amp^R^, RK2 origin, Xyls-Pm | (Wong and Mekalanos, 2000) |
| pSEVA228 | Km^R^, RK2 origin, Xyls-Pm | (Silva-Rocha et al., 2013) |
| pSEVA228-sfGFP | pSEVA228 ligated with sfGFP gene for recombinant protein expression | This work |
| pSEVA258 | Km^R^, RSF1010 origin, Xyls-Pm | (Silva-Rocha et al., 2013) |
| pSEVA258-sfGFP | pSEVA258 ligated with sfGFP gene for recombinant protein expression | This work |
| pSEVA234 | Km^R^, pBBR1 origin, LacIq-Ptrc | (Silva-Rocha et al., 2013) |
| pSEVA234-sfGFP | pSEVA234 ligated with sfGFP gene for recombinant protein expression | This work |
| pSEVA234-fleQ | pSEVA234 ligated with *fleQ* gene for overexpression of FleQ | This work |
| pSEVA658-sfGFP | pSEVA658 ligated with sfGFP gene for recombinant protein expression | (Yi et al,  in preparation) |

**Supplementary Table S2. Primers used in this study**

| Primer | Sequence (5'→3') |
| --- | --- |
| fleQ-TS1F | CGCGAATTCGAACTGGATCGCCTCGACAG |
| fleQ-TS1R | GCGAACACCCAGCCCAAACAATTTATGGCGCCGAAGCGTT |
| sfGFP-F | CGCGGATCCTACTAGAGAAAGAGGAGAAATACTAGATGGCAAGCAAGGGCGAGGA |
| sfGFP-R | CGCAAGCTTTTACTTGTACAGCTCGTCCA |
| fleQ-TS2F | TGTTTGGGCTGGGTGTTCGC |
| fleQ-TS2R | CGCGGATCCCACCCCGCCAGGCAGCAAGT |
| fleQ-comF | CGCGAATTCTACTAGAGAAAGAGGAGAAATACTAGATGTGGCGTGAAACCAAGAT |
| fleQ-comR | GGATCCTCAATCCTCCGCCTGGTCCT |

**Supplementary figure legends**

**Figure S1: ATP levels in *P. putida* KT2440 Wild Type and flagella-deleted strains.** The ATP bioluminescence assay was conducted using a commercially available kit as addressed in the method section following the manufacturer's protocol. Cells grown to mid-exponential phase in LB rich media and M9 minimal media containing succinate or glucose were assessed. Luminescence signals were measured using a microplate reader, and the obtained values were changed into nM units by standard curve and normalized to OD600 nm. The experiment was performed in three replicates. Statistical analysis (Student’s t-test) revealed no significant differences between the two strains.

**Figure S2. Difference in the resistance in chloramphenicol resistance between wild type and *fleQ*-deleted strains.** LB agar plates were prepared with varying Chl concentrations (0, 25, 75, and 175 μg ml^-1^). Serial dilutions of exponentially grown cells (ranging from10^−1^~10^−6^) were spotted onto plates that were incubated overnight and subsequently photographed.

**Figure S3. Measurement of the gene expression capacity using an orthogonal protomer.** The reporter gene was cloned into the pSEVA234 vector carrying the LacI^q^-Ptrc module (upper panel), and the resulting plasmid was introduced into both the wild-type (WT) and the *fleQ*-deleted strain. The reporter cells were cultured either in LB or in M9 minimal media containing 0.2% succinate for overnight. 500-fold diluted reporter strains were incubated in the same media for 13 h at 30 °C within a microplate reader. The expression levels of the fluorescent reporter were normalized to cell density and the maximum intensities of sfGFP at the 13-h time point. **p*,***p* ≤ 0.05 (Student’s t-test).

**Figure S4. Effect of nutrient quality on the expression of heterologous proteins expressed from a low copy number plasmid.** The low copy number plasmid (RK2 origin of replication), pSEVA228::sfGFP, was introduced into both the WT and the *fleQ*-deleted strains. (A) The resulting reporter strains were cultured in either LB (upper panel) or M9 media containing 0.2 % succinate (lower panel), and their growth was monitored. (B) To induce sfGFP expression, cultures described in (A) were supplemented with either 0.5 or 5 mM 3MBz. Normalized intensities of the reporter protein were evaluated, and the maximum value under each culture condition was presented. Error bars represent the standard deviation. Error bars represent means ± SD. (N = 3). **p*,***p*, ****p* ≤ 0.05 assessed using Student’s t-test.

**Figure S5. Investigation into the pleiotropic effect of *fleQ* on gene expression.** (A) Both the WT and the Δﬂagella strain, carrying the reporter plasmid and the *fleQ* expression plasmid, were grown at 30 °C for 13h in either LB medium or M9 + succinate (0.2 %) with 3MBz (5 mM) and IPTG (0.1 mM) in wells of a 24-well microplate. Growth was monitored using a microplate reader. (B) Fluorescence activities were measured at 13-h time point for each experimental condition. Error bars represent the standard deviations (N = 3). **p*≤ 0.005 ,** ≤ 0.05 assessed respectively, using Student’s t-test.

**References**

Grant, S.G., Jessee, J., Bloom, F.R., and Hanahan, D. (1990) Differential plasmid rescue from transgenic mouse DNAs into *Escherichia coli* methylation-restriction mutants. *Proc Natl Acad Sci U S A* **87**: 4645-4649.

Martinez-Garcia, E., and de Lorenzo, V. (2011) Engineering multiple genomic deletions in Gram-negative bacteria: analysis of the multi-resistant antibiotic profile of *Pseudomonas putida* KT2440. *Environ Microbiol* **13**: 2702-2716.

Martinez-Garcia, E., Nikel, P.I., Chavarria, M., and de Lorenzo, V. (2014) The metabolic cost of flagellar motion in *Pseudomonas putida* KT2440. *Environ Microbiol* **16**: 291-303.

Miller, V.L., and Mekalanos, J.J. (1988) A novel suicide vector and its use in construction of insertion mutations: osmoregulation of outer membrane proteins and virulence determinants in *Vibrio cholerae* requires toxR. *J Bacteriol* **170**: 2575-2583.

Nelson, K.E., Weinel, C., Paulsen, I.T., Dodson, R.J., Hilbert, H., Martins dos Santos, V.A. et al. (2002) Complete genome sequence and comparative analysis of the metabolically versatile *Pseudomonas putida* KT2440. *Environ Microbiol* **4**: 799-808.

Silva-Rocha, R., Martinez-Garcia, E., Calles, B., Chavarria, M., Arce-Rodriguez, A., de Las Heras, A. et al. (2013) The Standard European Vector Architecture (SEVA): a coherent platform for the analysis and deployment of complex prokaryotic phenotypes. *Nucleic Acids Res* **41**: D666-675.

Wong, S.M., and Mekalanos, J.J. (2000) Genetic footprinting with mariner-based transposition in *Pseudomonas aeruginosa.* *Proceedings of the National Academy of Sciences of the United States of America* **97**: 10191-10196.
