# Supplementary figures and images for: Impact of fleQ Deficiency on Resource Allocation and Heterologous Gene Expression in Pseudomonas putida Across Various Growth Media
Source: Microb Biotechnol. 2024 Nov 21;17(11):e70054. doi: 10.1111/1751-7915.70054 (PMC11580810; doi:10.1111/1751-7915.70054)

Figure S1

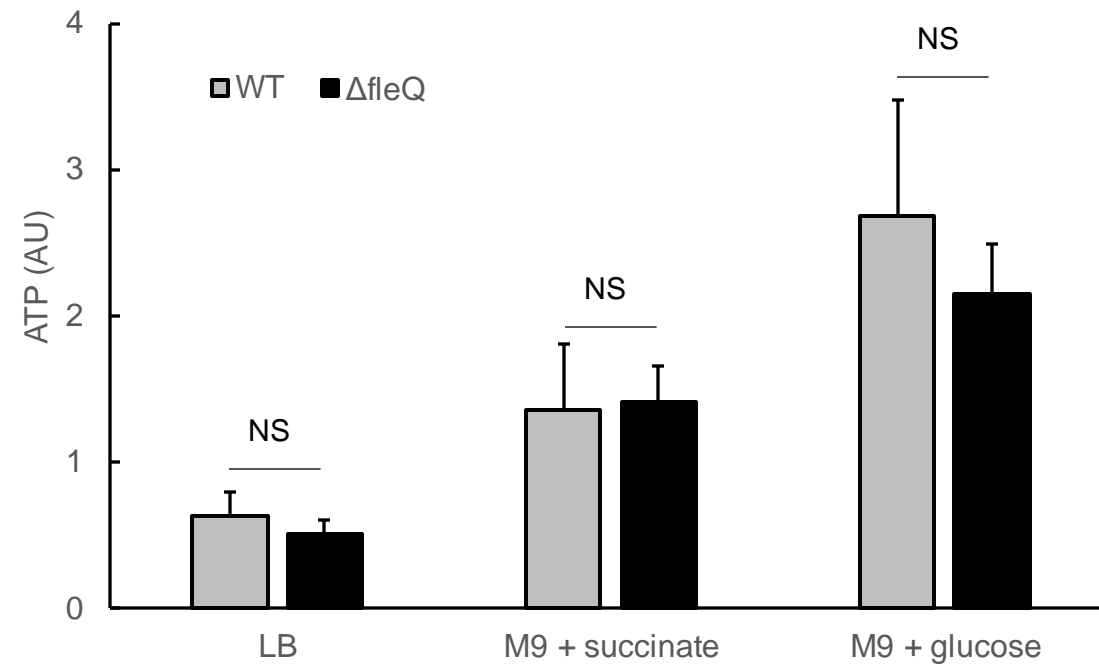

Figure S2

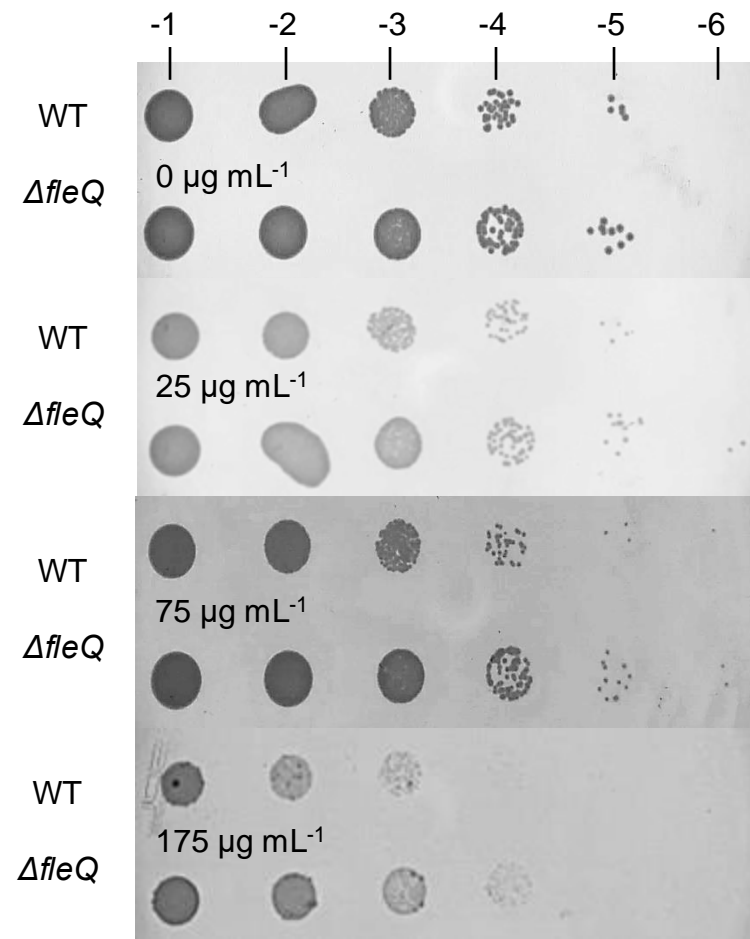

Figure S3

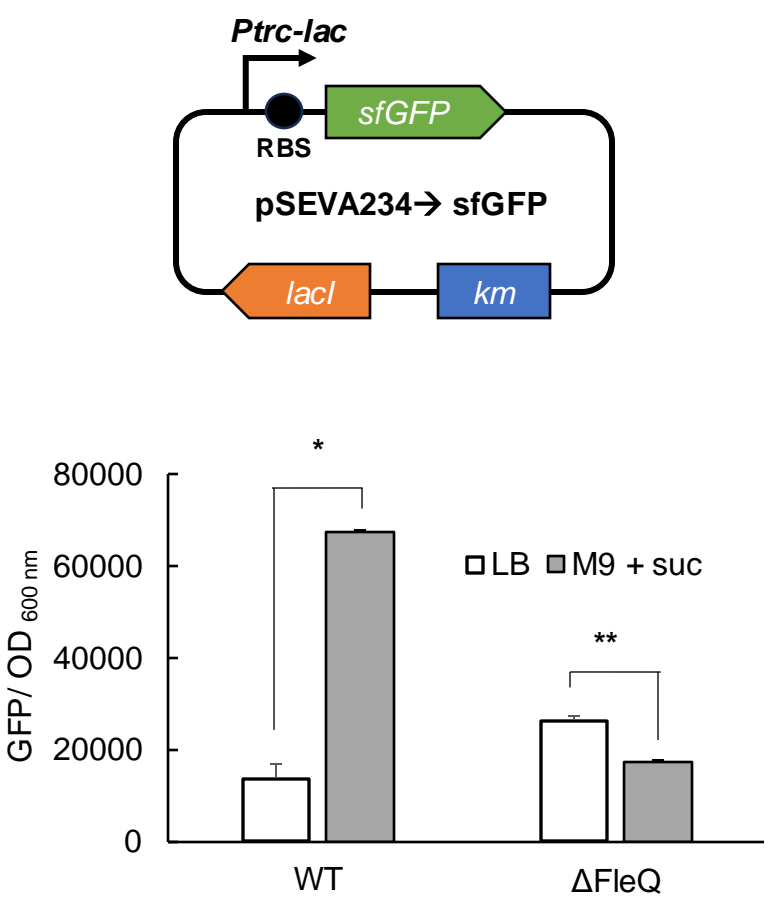

Figure S4

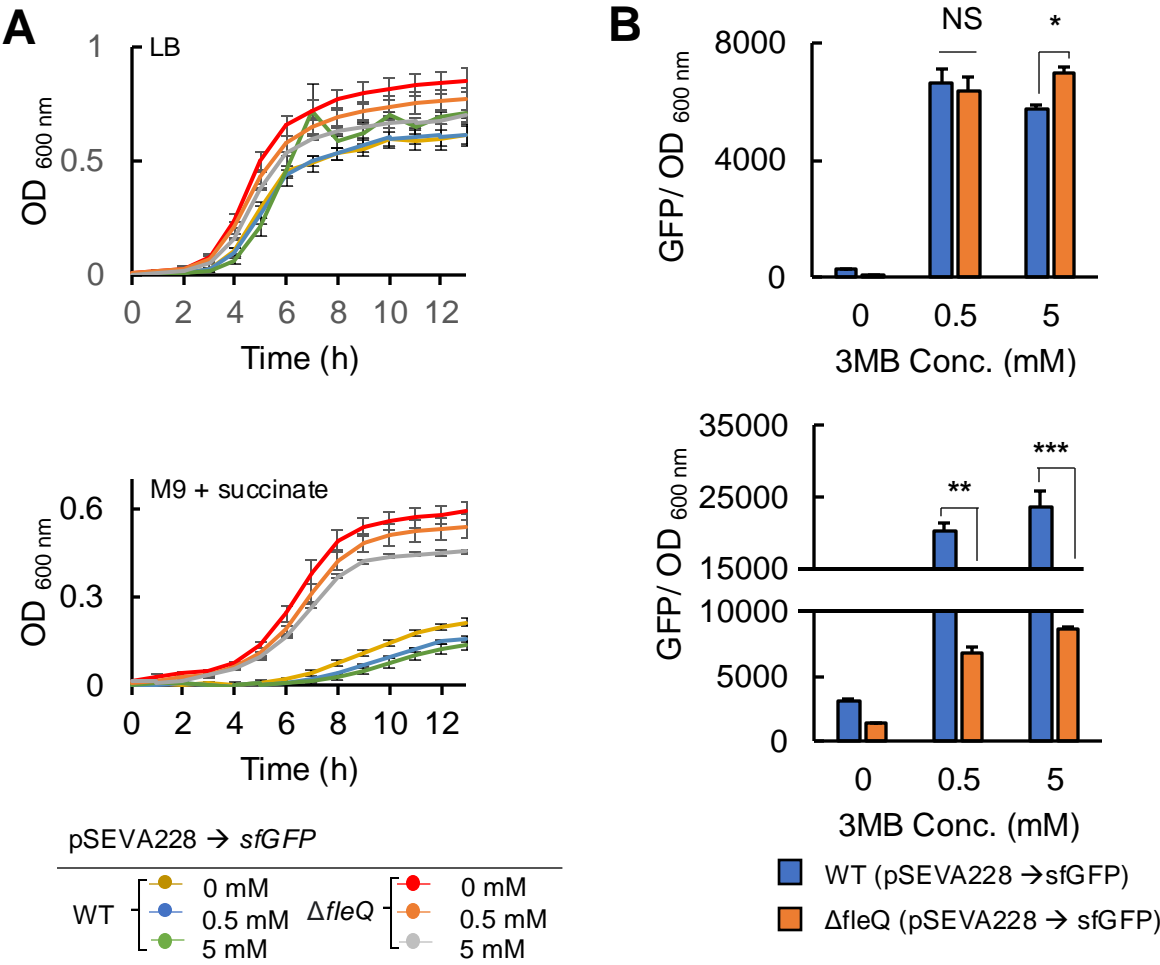

Figure S5

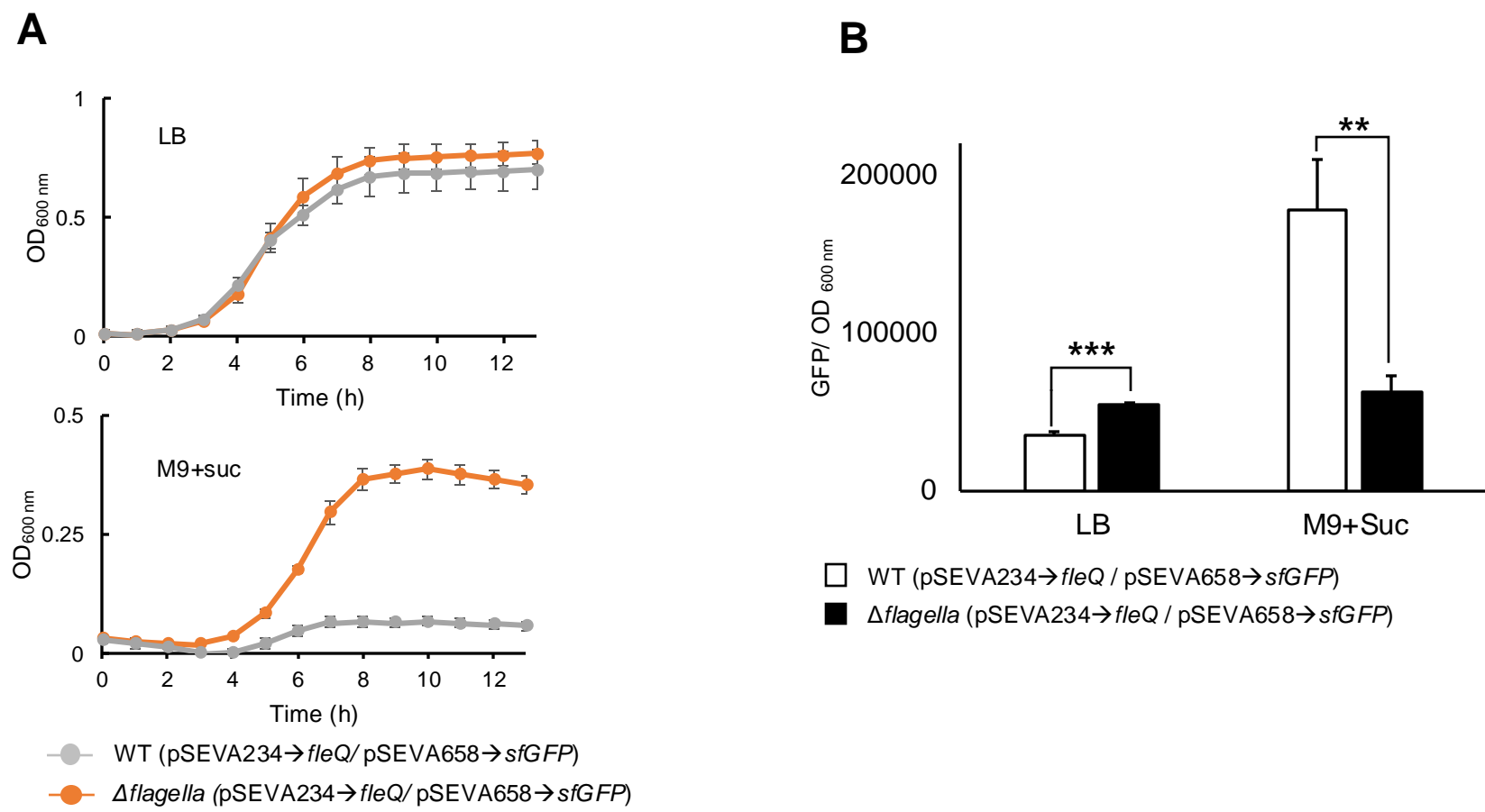

Supplement: Supplementary file 2 — Supporting Information S2. [file MBT2-17-e70054-s001.pdf]
